# Anti-obesity Medication Use for Adolescent Metabolic and Bariatric Surgery Patients: A Systematic Literature Review

**DOI:** 10.7759/cureus.50905

**Published:** 2023-12-21

**Authors:** William N Doyle, Nolan Reinhart, Nikhil C Reddy, Abdul-Rahman F Diab, Joseph A Sujka, Christopher G DuCoin, Salvatore Docimo

**Affiliations:** 1 Department of Surgery, University of South Florida Morsani College of Medicine, Tampa, USA

**Keywords:** anti-obesity medications, bariatric surgery, bariatric pediatric surgery, weight loss medications, adolescent obesity

## Abstract

Bariatric surgery, in combination with pharmacotherapy, has been proven to be successful in combatting weight regain in adults; however, the use of anti-obesity medications to augment weight loss in adolescents before and after bariatric surgery is not well studied. In adolescent obese patients, the efficacy of anti-obesity pharmacotherapy before and after bariatric surgery on weight loss compared to no interventions in various studies was investigated.

A PubMed literature search using the Preferred Reporting Items for Systematic Reviews and Meta-Analyses (PRISMA) guidelines was performed to identify studies related to the pharmacologic treatment of obesity in adolescents with a history of bariatric surgery. Inclusion criteria consisted of clinical trials, case reports, case series, chart reviews, systematic reviews, and meta-analyses written in English and published between 2005 and 2022 using our search criteria. Exclusion criteria were studies that investigated adults, did not include pharmacotherapy, and were not relevant to the outcome of interest.

The initial search yielded 1275 results, which was reduced to 879 after removal of duplicates. After applying exclusion criteria, the number of articles was reduced to 63. Full articles were examined and 44 were excluded due to relevance. Nineteen articles were included in the qualitative analysis. A total of 2471 adolescents were treated with various types of pharmacotherapy, 65 of whom had a history of bariatric surgery. The results showed varied effects of pharmacotherapy with the different medications studied. However, the 65 patients were included in cohorts of patients with no history of bariatric surgery. These studies did not include data specific to adolescent bariatric surgery patients.

There is a wealth of evidence highlighting the efficacy of pharmacotherapy in assisting with weight loss in adolescents with obesity; however, our literature search showed a lack of studies focusing on the use of pharmacotherapy in the adolescent bariatric surgery population. Potential limitations include missing studies in our literature search, the variability in methods between studies, and the lack of standardized quality assessment. Additionally, studies involving our objective of choice regarding bariatric surgery with anti-obesity medication were limited. Clinical trials to determine the efficacy of medications as an adjunct to bariatric surgery in preventing weight regain and leading to optimal weight loss in this population are of utmost importance.

## Introduction and background

Obesity is common in the United States, affecting more than 40% of Americans in 2020 [1]. Obesity-associated comorbidities such as heart disease, type 2 diabetes mellitus (T2D), and stroke lead to decreased quality of life and many preventable hospitalizations and deaths. The obesity epidemic is also impacting adolescent populations, with 22.2% of children aged 12-19 living with obesity, measured as a BMI greater than the 95th percentile for their age [1]. In addition to the negative health effects of obesity, childhood obesity can severely impair psychosocial health in a critical developmental period causing increased levels of stress, depression, and decreased resilience in youth [2]. Physicians can combat adolescent obesity by employing lifestyle modification therapy along with pharmacotherapy and even bariatric surgery. Adolescents generally qualify for bariatric surgery when a trial of lifestyle modification and weight loss medication fails. 

There is a lack of clinical guidelines pertaining to anti-obesity medication options to augment the treatment of adolescent obesity in combination with bariatric surgery. However, pharmacotherapy in conjunction with bariatric surgery is well-studied in adult patients. A recent review of literature from the American Society for Metabolic and Bariatric Surgery highlighted how anti-obesity medications (AOMs) can be used effectively for adults preoperatively as well as postoperatively in patients with weight recurrence [3]. This review also calls for the further study of AOM usage for adolescent patients, especially in the postoperative period to manage weight regain. As the volume of these procedures increases in adolescents, more data is needed to evaluate outcomes and clinical guidelines. 

The objective of this literature review was to examine the efficacy of various weight loss medications in adolescent obese patients in the setting of bariatric surgery. In our review, we found multiple weight loss medications studied without bariatric surgery that have been recently shown to have great efficacy in adolescents as well as point out the need for further research into the usage of anti-obesity medications in the adolescent bariatric surgery population. 

## Review

Methods 

Three reviewers worked together to perform a literature search in PubMed to identify studies pertaining to the pharmacologic treatment of obesity in adolescent bariatric surgery patients. Search terms input through PubMed included “adolescent anti-obesity medication,” “adolescent AND weight loss medication,” “anti-obesity medication AND adolescent AND bariatric,” “pediatric anti-obesity medication,” “anti-obesity medication AND pediatric AND bariatric,” “anti-obesity medication AND pediatric AND bariatric surgery.” We utilized the Preferred Reporting Items for Systematic Reviews and Meta-Analyses (PRISMA) guidelines to identify publications of interest. The inclusion of studies was limited to clinical trials, case reports, case series, chart reviews, systematic reviews, and meta-analyses written in English and published between 2005 and 2022. We also identified articles from the references of the articles from our search results to expand our list of publications. Institutional review board approval was not required because the study is a review of current literature and does not involve the participation of patients. We filtered the publications that described the usage of anti-obesity medications in adolescent patients with mention of adolescent bariatric patients. Studies focusing on the use of anti-obesity medications in adolescents date back to 2005; therefore, studies published before 2005 were excluded. Those not directly relevant to our outcome of interest were also excluded. 

Results 

**Figure 1:**
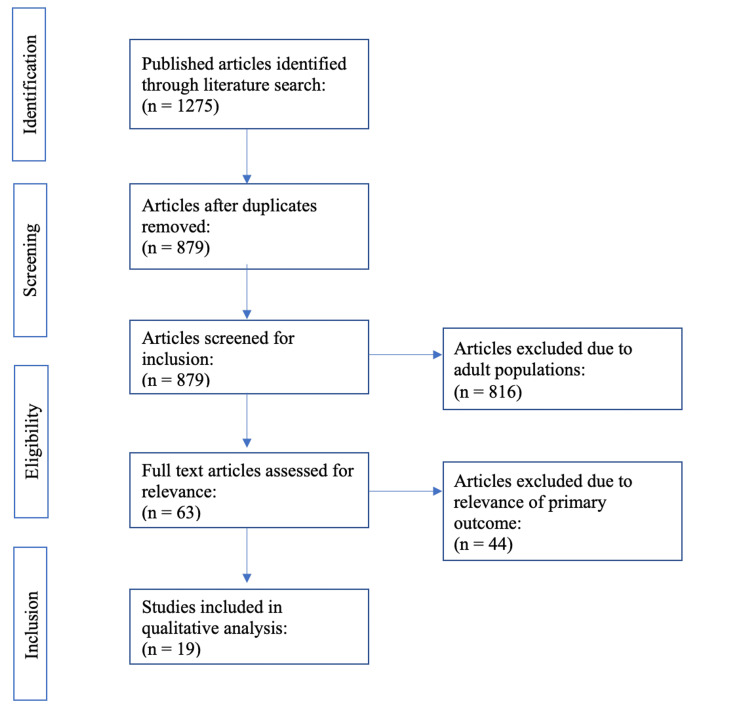
PRISMA diagram Figure 1: Preferred Reporting Items for Systematic Reviews and Meta-Analyses (PRISMA) diagram highlighting the system utilized to narrow down potential sources for the review of literature.

As seen in Figure 1, the initial database search yielded 1275 results. Following the removal of duplicates, 879 publications remained. This was reduced to 63 sorted for our exclusion criteria of adult populations by reading titles. Full articles were then examined and 44 were excluded due to relevance to our primary outcome of interest: combined weight loss due to anti-obesity medications and bariatric surgery in adolescent patients. Nineteen articles were included in our qualitative analysis. A summary of patient populations and pharmacotherapy for these 19 studies is included in Table 1. A total of 2471 adolescents were treated with pharmacotherapy, 65 of whom had a history of bariatric surgery. However, none of these studies reported weight loss data specific to these bariatric patients. 

**Table 1:** Review of 19 included studies Summary of the patient populations of the 19 studies included in the review [4-22]. Exenatide XR: exenatide extended-release; Metformin XR: metformin extended-release

Study Name	Publication Year	Mean Age (Range)	n	Pharmacotherapy
Effect of Orlistat on Weight and Body Composition in Obese Adolescents: A Randomized Controlled Trial	2005	13.6 (12-16)	539	Orlistat
Randomized, Double-Blind, Placebo-Controlled Trial of Orlistat for Weight Loss in Adolescents	2006	15.8 (14-18)	40	Orlistat
Effect of Phentermine on Weight Reduction in a Pediatric Weight Management Clinic	2018	16.1 (12-18)	299	Phentermine
Topiramate for Weight Reduction in Adolescents with Severe Obesity	2015	15.2 (up to 18)	28	Topiramate
Meal Replacements followed by Topiramate for the Treatment of Adolescent Severe Obesity: A Pilot Randomized Controlled Trial	2016	15.2 (12-18)	21	Topiramate
The Potential Role of Combination Pharmacotherapy to Improve Outcomes of Pediatric Obesity: A Case Report and Discussion	2018	10	1	Phentermine/topiramate
A Randomized, Double-Blind, Placebo-Controlled, Pharmacokinetic and Pharmacodynamic Study of a Fixed-Dose Combination of Phentermine/Topiramate in Adolescents with Obesity	2019	(12-17)	42	Phentermine/topiramate
Phentermine/Topiramate for the Treatment of Adolescent Obesity	2022	14.0 (12-17)	223	Phentermine/topiramate
Metformin Use in Children with Obesity and Normal Glucose Tolerance--Effects on Cardiovascular Markers and Intrahepatic Fat	2012	12.3 (8-17)	42	Metformin
Metformin in Obese Children and Adolescents: the MOCA Trial	2013	13.7 (8.25-18.42)	151	Metformin
Effects of Metformin on Body Weight and Body Composition in Obese Insulin Resistant Children: A Randomized Clinical Trial.	2011	10.1 (6-12)	100	Metformin
Metformin Extended Release Treatment of Adolescent Obesity: a 48-week Randomized, Double-Blind, Placebo-Controlled Trial with 48-week Follow-up	2010	14.8 (13-18)	77	Metformin XR
Exenatide as a Weight-Loss Therapy in Extreme Pediatric Obesity: A Randomized, Controlled Pilot Study	2012	12.7 (9-16)	12	Exenatide
The Effect of Glucagon-Like Peptide-1 Receptor Agonist Therapy on Body Mass Index in Adolescents with Severe Obesity: a Randomized, Placebo-Controlled, Clinical Trial	2013	15.2 (12-19)	22	Exenatide
A 6-month Randomized, Double-Blind, Placebo-Controlled Trial of Weekly Exenatide in Adolescents with Obesity	2020	14.5 (10-18)	44	Exenatide XR
Exenatide for Weight-Loss Maintenance in Adolescents with Severe Obesity: A Randomized, Placebo-Controlled Trial	2022	16.0 (12-18)	66	Exenatide XR
Liraglutide in an Adolescent Population with Obesity: A Randomized, Double-Blind, Placebo-Controlled 5-Week Trial to Assess Safety, Tolerability, and Pharmacokinetics of Liraglutide in Adolescents Aged 12-17 Years	2017	15.1 (12-17)	21	Liraglutide
A Randomized, Controlled Trial of Liraglutide for Adolescents with Obesity	2020	14.6 (12-18)	251	Liraglutide
Pharmacotherapy for the Treatment of Overweight and Obesity in Children, Adolescents, and Young Adults in a Large Health System in the US	2020	15.8 (13-18)	492	Metformin, topiramate, bupropion, phentermine, zonisamide

*Orlistat * 

Orlistat is the only weight-loss medication approved by the FDA for long-term use in adolescents with obesity. It has been approved for use in adolescents ≥ 12 years old [23]. The typical prescribed dosage is 120mg three times daily taken with meals [23]. Our literature review yielded two older randomized controlled trials that proposed its efficacy in treating adolescent obesity. 

The first trial published in 2005 was a multicenter, placebo-controlled study that compared outcomes of adolescents aged 12-16 taking 120mg of orlistat three times daily to placebo. At the end of the 54 weeks, the orlistat group experienced a mean BMI change of -0.55kg/m2, while the placebo group showed a change of + 0.31kg/m2 (p<0.001) [4]. Compared with 15.7% of the placebo patients, 26.5% of the orlistat-treated patients experienced at least a 5% BMI reduction. Furthermore, 13.3% of the orlistat treated patients, and only 4.5% of the placebo group experienced a BMI reduction of 10% or greater. Orlistat-treated patients experienced steady weight loss until week 12 when weight loss plateaued. Both groups experienced weight regain toward the end of the study [4]. 

Another randomized, placebo-controlled trial study published a year later in 2006 also compared the effects of orlistat 120mg three times daily to placebo. There was no statistically significant difference between the groups at the end of the six-month study period with respect to BMI change. The orlistat group experienced an average BMI change of -1.3kg/m2 compared to the placebo group’s -0.83kg/m2 change (p=0.39) [5]. There was also no significant difference in average weight change between the orlistat group (-5.5kg) and the placebo group (-1.6kg) (p=0.13) at the six-month follow-up [5]. The orlistat group experienced more adverse events, primarily related to bowel movements, than the placebo group [5]. 

Phentermine 

Phentermine is a norepinephrine reuptake inhibitor that is approved by the FDA for use in adolescents > 16 years old, however, only up to a 12-week duration. The typically prescribed dose is either 15mg, 30mg, or 37.5 mg per day [23]. Phentermine is the most prescribed medication to treat obesity in the adult population; however, it has been studied very little in adolescents [6]. 

A retrospective chart review published in 2017 compared the weight loss efficacy of phentermine 15mg daily in addition to standard of care lifestyle modification therapy (SOC) versus SOC alone in a cohort of adolescents with a mean age of 16.1 years old. Phentermine use was associated with a statistically significant greater % change in BMI at one, three, and six months when compared to SOC alone. A -1.6% change in BMI was observed at one month, a -2.9% change was observed at three months, and a -4.1% change in BMI was observed at six months [6]. Significant reductions in weight and absolute BMI were also observed at one month (-1.4kg, -0.6kg/m2), three months (-2.6kg, -1.1kg/m2), and six months (-3.2kg, -1.6kg/m2) in the phentermine plus SOC group compared to the SOC-only control group [6]. At three months, 40% of the phentermine group experienced a clinically significant BMI reduction of ≥ 5%, compared to only 8.8% in the SOC-only group. At six months, these proportions grew to 63.6% and 20.8%, respectively [6]. The only statistically significant adverse reaction that was noted in this review was an increase in heart rate amongst the phentermine plus SOC patients at three-month follow-up [6]. This heart rate elevation was not significant at one- or six-month follow-up. 

A retrospective clinical database study published in 2020 analyzed weight loss in adolescents on multiple different medications including phentermine, topiramate, bupropion, metformin, and zonisamide. Researchers found that adolescents on phentermine experienced greater average weight loss over the course of the study period than any other medications, with a mean decrease of 0.12% of total body weight (TBW), which is statistically significant when compared with the overall frequency-weighted mean of the study cohort [7]. Every other drug included in this study led to weight gain in adolescents. Patients on metformin had a mean increase in TBW of 0.01%, patients on topiramate had a mean increase in TBW of 0.08%, patients on bupropion had a mean increase in TBW of 0.04%, and patients on zonisamide had a mean increase in TBW of 0.08% [7]. 

Topiramate 

Topiramate has not been approved by the FDA for the treatment of obesity in adolescents [23]. However, some use it off-label. The recommended dosages range from 25mg to 100mg [23]. Our literature review yielded a retrospective chart review investigating the efficacy of topiramate in an adolescent population [8] as well as a follow-up randomized controlled trial examining if meal-replacement therapy before topiramate initiation in adolescents is effective in combatting obesity [9]. 

Researchers from the University of Minnesota (Minneapolis, Minnesota, United States) found topiramate (75 mg daily), along with lifestyle modification received at the weight management clinic, to be efficacious in treating obesity in a group of adolescents analyzed retrospectively. At three and six-month follow-ups, the topiramate group experienced a -3.9 and -6.6 mean % change in BMI, respectively. The group not undergoing topiramate treatment also experienced a statistically significant % BMI reduction, but not to this extent [8]. The topiramate group had a greater proportion of patients experiencing BMI reduction of 5% or more at three-month follow-up (27.3% vs. 17.9%) and six-month follow-up (66.7% vs. 50.0%). However, at the six-month follow-up, the group not treated with topiramate had a greater proportion of patients experience a BMI reduction of 10% or more (13.6% vs. 11.1% in the topiramate group) [8]. 

This same group of researchers completed a follow-up randomized controlled trial one year later. They found that a 24-week regimen of 75mg topiramate daily following four weeks of meal replacement therapy demonstrated limited success in reducing BMI in adolescents compared to placebo following meal replacement therapy [9]. Both the above studies noted patients experiencing paresthesia while prescribed topiramate. 

Phentermine + Topiramate (PHEN/TPM)

PHEN/TPM extended-release (XR) combination treatment for obesity has been found to achieve greater weight loss than either medication alone. This treatment is currently FDA-approved for long-term use for adults ≥ 18 years old. 

A case report published in 2018 was the first research to show the efficacy of PHEN/TPM dual therapy for pediatric patients [10]. This case report highlights the experience of a 10-year-old girl who initially experienced a 15% reduction in BMI through lifestyle modification therapy. Within two years, her BMI returned to baseline. The patient, BMI of 34.1kg/m2 at the time, was then treated with TPM 75mg and PHEN 15mg for 22 months, and the patient’s BMI was reduced to 25.7kg/m2. This patient’s success with PHEN/TPM combination therapy paved the way for randomized controlled trials analyzing a larger adolescent population [10]. 

A randomized controlled trial published in 2019 tested two different dosing regimens of PHEN/TPM over 56 days in adolescent patients between the ages of 12-17. At day 56, adolescents treated with PHEN/TPM 15mg/92mg experienced a mean % weight change of -4.96%. This compares to a change of -3.72% for those treated with PHEN/TPM 7.5mg/46mg and a change of +1.06% for those in the placebo group [11]. The percentage of adolescents that experienced at least 5% weight loss at day 56 was 50.0% in the PHEN/TPM 15mg/92mg group, 13.3% in the 7.5mg/46mg group, and 0.0% in the placebo group. The investigators determined that 35.7% of participants had a treatment-emergent adverse event related to PHEN/TPM [11]. 

A similar trial was published in 2022 with a greater number of participants and a greater follow-up of 56 weeks. The included participants had a mean age of 14.0. PHEN/TPM 15mg/92mg was categorized as top-dose and PHEN/TPM 7.5mg/46mg was categorized as mid-dose [12]. In regard to weight change, participants treated with high-dose PHEN/TPM experienced a mean weight change of -9.23kg, compared to -5.49kg in the mid-dose group, and +6.07kg in the placebo group. The top-dose group also experienced the largest mean % BMI change, with -7.11, followed by the mid-dose group at -4.78 [12]. The placebo group’s mean % BMI change was +3.34%. The top-dose group had the largest proportion of patients experiencing a reduction of BMI by at least 5%, 10%, and 15%. The mid-dose group and placebo group had the second largest and smallest proportion of patients reach these benchmarks, respectively. The proportion of participants receiving top-dose treatment who had a treatment-emergent adverse event was 52.2%, followed by the placebo group at 51.8%, then the mid-dose group at 37.0% [12]. 

Metformin 

Metformin is another off-label medication often used to treat obesity in adolescents, despite the mechanism for its weight loss efficacy being unknown. Metformin is FDA-approved to treat T2D in pediatric patients > 10 years old [23]. The recommended dosage ranges between 500-2000mg divided twice daily [23]. 

A randomized controlled trial published in 2010 examined the efficacy of metformin hydrochloride XR in adolescents aged 13-18 years old by comparing outcomes of a metformin-treated cohort to a placebo cohort over 48 weeks. At the end of the study, the mean adjusted BMI increased by 0.2 kg/m2 for the placebo group and decreased by 0.9 kg/m2 for the metformin XR-treated patients. These results were statistically significant. This difference in BMI remained for 12 to 24 weeks after treatment and lifestyle intervention programs were stopped [13]. 

Another randomized controlled trial, this one published in 2011, also analyzed the effects of metformin therapy on weight and body composition. The age group included in this study was children between the ages of six and 12 years. The participants were randomized to either metformin 1000mg twice daily or placebo, for six months [14]. Children treated with metformin had a statistically significant greater mean BMI reduction (-0.78kg/m2 vs. +0.32kg/m2 in the placebo group). While both groups experienced an increase in weight at the end of the trial, the metformin group’s mean weight gain was significantly less (1.47kg) when compared to the placebo group (4.85kg) [14]. 

A randomized control trial published in 2012 set out with the aim of identifying the effect of metformin on cardiovascular markers and intrahepatic fat in children between the ages of seven and 18. This study also measured weight loss and BMI reduction at the end of the six-month intervention period [15]. The metformin-treated group (mean age of 12.3) experienced a significantly greater mean weight change of -4.9kg when compared to the diet and exercise intervention group (mean age 12.0), which experienced a mean weight change of -1.7kg. A significantly greater mean BMI reduction was also observed in the metformin-treated patients (-2.4kg/m2) compared to the diet and exercise-only group (-1.1kg/m2) [15]. 

The Metformin in Obese Children and Adolescents (MOCA) trial was a prospective, randomized controlled trial to assess the impact of metformin on body composition and other metabolic factors. The trial included participants between the ages of eight and 18, with the mean age of participants being 13.7 years old. The intervention group was put on a regimen of 1000mg metformin in the morning and 500mg metformin in the evening. The participants treated with metformin experienced a significantly greater reduction in BMI compared to the placebo group at both three and six-month follow-ups [16]. 

Glucagon-Like Peptide 1 (GLP-1) Analogs 

GLP-1 acts mainly as an incretin, promoting postprandial insulin release and improving pancreatic β-cell function, and has also been reported to inhibit food intake in humans. It has been shown that Roux-en-Y gastric bypass (RYGB) surgery increases the levels of GLP-1 release post-prandially in humans, so there could possibly be a synergistic effect with the addition of GLP-1 analogs as adjunctive therapy in adolescents post-bariatric surgery [24]. These medications are used off-label for weight loss in adults with the exception of liraglutide, which is FDA-approved for long-term use in adults for the purpose of weight loss. There are a few studies examining GLP-1 agonists in adolescents, the most extensively studied in the adolescent population being exenatide. 

A randomized controlled trial published in 2012 served as a pilot study for exenatide in the pediatric population with obesity including 12 subjects aged nine to 16. It was an open-label trial with a three-month study phase and a three-month control phase. The study group was started on exenatide 5mcg subcutaneous injection twice a day and titrated up to 10mcg after one month. The study group showed a significantly greater reduction in BMI (-0.9kg/m2) than control (+0.84kg/m2). Side effects reported include mild gastrointestinal symptoms [17]. 

A follow-up study by the same group in 2013 investigated longer-term use of exenatide in adolescents. Twenty-six patients aged 12-19 with severe obesity participated in a three-month randomized controlled trial followed by three months of open-label extension for a total of six months of treatment. The same dosing scheme was used in this study, with 5mcg twice daily titrated up to 10mcg twice daily after one month. Again, the treatment group saw a significantly greater decrease in BMI (-1.13kg/m2 compared to placebo after three months, -1.67kg/m2 after six months compared to placebo). The side effect profile continued to be acceptable with mild gastrointestinal discomfort [18]. 

Another study published in 2020 investigated exenatide XR weekly injections in adolescents aged 10-18. Forty-four participants were enrolled for a six-month randomized, double-blind placebo-controlled trial of exenatide XR 2mg weekly plus lifestyle modification therapy vs. placebo (lifestyle modification therapy alone). This study found a significant 0.83 kg/m2 decrease in BMI vs. placebo, with the minimal side effects noted being gastrointestinal in nature (flatulence, nausea, diarrhea, constipation, abdominal pain, burping, vomiting, and mouth pain) [19]. 

The latest study looking at exenatide XR was published in 2022 and investigated the effectiveness of exenatide for weight loss maintenance. Sixty-six participants aged 12-18 who achieved ≥ 5% BMI reduction after four to eight weeks of a meal-replacement therapy run-in phase were then randomized 1:1 into intervention and control groups for an additional 52 weeks. Both groups regained weight at the end of the study but the experimental group regained less weight (BMI increase of 2.7kg/m2 compared to starting BMI) than the control group (BMI increase of 9.6kg/m2 compared to starting BMI). Although these results were not quite significant (p=0.098), they show that exenatide treatment may help mitigate the rebound weight gain in adolescents after initial success in weight loss [20]. 

The pilot study for liraglutide use in adolescents, published in 2017, was a randomized double-blind controlled trial to assess the safety and dosage of liraglutide in adolescents. It enrolled patients aged 12-17 to randomly receive five weeks of treatment of 0.6mg with a weekly dose increase to a max of 3mg in the final week. Fourteen received the treatment and seven received the placebo. The primary endpoint was treatment emergent adverse events (TEAE). All treatment patients and four of seven placebo patients had at least one TEAE, which were most commonly gastrointestinal disorders with no severe TEAEs. This study shows that liraglutide has a similar safety profile in adolescents compared to adults with dosing regimens being similar with minimal safety issues [21]. 

A follow-up study was published in 2020, where researchers conducted a randomized double-blind trial that enrolled patients between age 12-18 with obesity and poor response to lifestyle changes into a 56-week treatment period of either subcutaneous liraglutide (3mg daily) or placebo. The primary endpoint was a change in BMI standard deviation at 56 weeks. There were 125 participants assigned to the experimental group and 126 to the placebo group. The liraglutide group had a significant BMI standard deviation change compared to the control (-0.22kg/m2). Liraglutide had a larger reduction in BMI compared to placebo (estimated difference was -4.64 %) and body weight (estimated difference was - 5.01 %) [22]. 

PHEN/TPM in Adolescents after Bariatric Surgery 

Our literature search yielded one ongoing pilot clinical trial investigating the effects of PHEN/TPM in adolescents with inadequate weight loss after bariatric surgery. The results of this study are not currently available. This was the only study found focusing on anti-obesity pharmacotherapy intervention in adolescents with a history of bariatric surgery. 

Discussion

Current treatment options for obesity in adolescent patients are multidisciplinary in nature, with a heavy emphasis in favor of lifestyle modifications, including dietary and exercise implementations. Over-emphasis on behavior and lifestyle change may precipitate psychological disorders, including anxiety, depression, low self-esteem, and eating disorders. These negative consequences can be exacerbated more when long-term weight loss is not successfully achieved [25]. There are only two medications currently approved by the FDA for the treatment of adolescent obesity. Clinicians often resort to obesity medications commonly prescribed to adults and prescribe them off-label to their adolescent patients. We sought to summarize the current landscape of anti-obesity pharmacotherapy for the adolescent population in this brief literature review. 

This study had several limitations. Although broad search terms were used in our search, studies outside the scope of our PubMed search may have been excluded. Additionally, there is a lack of standardization of doses and criteria for patient treatment across studies. We did not use a quality assessment tool to evaluate the strength of evidence for each study included. We were unable to explore our initial objective due to the lack of anti-obesity medications studied with the use of bariatric surgery. This absence of studies investigating the use of weight loss medications in the pre- and post-operative setting for adolescent bariatric surgery patients is evident and this literature review highlights an important lack of data. 

There is a wealth of research that highlights the efficacy of pharmacotherapy in assisting with the weight loss of adolescents with obesity, highlighted previously. Yet, our review of the literature showed a lack of research that focuses on specifically bariatric patients. This is concerning considering that now the American Academy of Pediatrics guidelines published on January 9th, 2023, advise primary care providers to offer adolescents 12 years and older with obesity (BMI ≥95th percentile) weight loss pharmacotherapy, according to medication indications, risks, and benefits, as an adjunct to health behavior and lifestyle treatment (Class B) and offer referral for adolescents 13 years and older with severe obesity (BMI ≥120% of the 95th percentile for age and sex) for evaluation for metabolic and bariatric surgery to local or regional comprehensive multidisciplinary pediatric metabolic and bariatric surgery centers (Class C) [26]. Further research into this topic is important as an increased number of adolescents elect to undergo weight loss surgery, now that it has been shown to be safe for the pediatric population [27]. Clinical trials into this population are of utmost importance to determine the efficacy of medications as an adjunctive treatment with bariatric surgery to prevent weight regain and lead to optimal weight loss in the setting of this ever-growing obesity epidemic in adolescents today. 

## Conclusions

The utilization of pharmacotherapy to augment weight loss before and after bariatric surgery is well-studied in the adult population and has been shown to prevent weight regain following bariatric surgery. Bariatric surgery has been proven to be safe for adolescents, and its usage is increasing in younger patients. Despite the safety and efficacy of bariatric surgery and pharmacotherapy for managing weight in adolescents with obesity, there is a lack of studies examining the utilization of both combined. There is a need for randomized controlled trials examining anti-obesity medications in adolescents with a prior history of bariatric surgery, especially considering they have shown to be efficacious for augmenting weight loss in adult bariatric patients.
